# Prioritizing Health Equity: Patient Perspectives from a Clinic-Based PhotoVoice Qualitative Study

**DOI:** 10.1089/heq.2019.0082

**Published:** 2019-11-04

**Authors:** Susi L. Keefe, Raie M. Gessesse, Emily R. Lincoln, Kyle Meerkins, Taylor R. Evans

**Affiliations:** ^1^ Public Health Sciences, Hamline University, St. Paul, Minnesota.; ^2^ Family Tree Clinic, St. Paul, Minnesota.

**Keywords:** health equity, PhotoVoice, community-engaged research, reproductive health

## Abstract

**Purpose:** This article explores the results of community-engaged PhotoVoice research with the Family Tree Clinic (FTC) in St. Paul, MN. FTC has >45 years of experience providing sexual, reproductive, and primary health care, with a central mission of overcoming issues for their patients including those of poverty, oppression, lack of access, and discrimination in meeting health care needs.

**Methods:** This research presents the findings of social justice-inspired PhotoVoice focus groups with patients of the clinic that asked two central questions: “Why do you choose Family Tree Clinic” and “What stands in the way of achieving your goals for your health?”

**Results:** When health equity is a central priority and evident in clinic culture, practices, and policies, patients articulate positive experiences despite real structural and systemic barriers outside the clinic.

**Conclusion:** We offer suggestions for a health equity-oriented approach to clinic care.

## Introduction

Health equity is the most central theoretical and applied concept guiding global and public health today.^1–4^ Together with Family Tree Clinic (FTC) we explored how patients experience clinic care when health equity is applied as a motivating theoretical concept. In this clinic, health equity is not just a concept articulated in the mission statement, but it is also integrated into every aspect of clinic policy, structure, and practice.

The clinic's roots in the women's health movement launched access to contraception and high-quality confidential sexual health care in an urban setting in the 1960s, and since has expanded its expertise to LGBTQ+ health care. Research on distinctly LGBTQ+ populations continues to be limited, but the unique health experiences and disparities are well established.^5–7^ “Although the acronym LGBT is used as an umbrella term, and the health needs of this community are often grouped together, each of these letters represents a distinct population with its own health concerns.”^7 (p.1)^ To address the health needs and disparities of LGBTQ+ communities, research and clinical approaches to LGBTQ+ health care need to acknowledge “(a) heterogeneity and intersectionality within LGBT communities; (b) the influence of structural and environmental context; and (c) both health-promoting and adverse pathways that encompass behavioral, social, psychological, and biological processes.”^6 (p.653)^

In 2014, FTC formed a work group to be more deliberate about justice in their core values. FTC core values exist around four key areas: commitment to furthering social and reproductive justice, mission focused, collaborative and accountable, and direct and respectful actions (Family Tree Clinic Employee Handbook, 2016, unpublished data). These values put into practice a conscious awareness of cultural markers and paradigms and created a workplace environment that is in service to the mission. In addition, these values allowed FTC staff to be self-aware of mistakes made and commit to a culture of constantly learning and evolving (Family Tree Clinic Employee Handbook, 2016, unpublished data).

Today, FTC has a renewed commitment to reproductive justice of marginalized communities more broadly. Reproductive justice utilizes an intersectional approach of human rights and identifies reproductive oppression as the primary problem. Reproductive rights, until recently, failed to acknowledge the history of reproductive oppression, the struggles of women of color, structural systems of power, and other significant factors that confound our ability to accept the reproductive rights framework as sufficient.^8,9^ Drawing from intersectional theory, FTC identifies and locates intersecting social constructions of power such as racism, sexism, classism, ageism, ableism, nativity and ethnicity, and citizenship status.^10–13^ Intersectionality, both as a theory and methodological approach, becomes essential to the delivery of care in ways that are critical to the elimination of health disparities.

The aim of this PhotoVoice focus group study was to highlight how patients experience health equity approaches at a clinic that prioritizes reproductive justice, LGBTQ+ core competencies, and intersectionality in clinical care.

## Methods

### Ethical considerations

The Institutional Review Board at Hamline University approved the research study. All participants in the focus groups and interviews signed our consent form before participating. All pictures were taken by participants and are used with permission.

### Setting/recruitment/sample

Fourteen students in an advanced health equity course engaged in ethnographic participant observation at the clinic and spent 4 h per week for 7 weeks collecting and recording data, which resulted in a total of 392 clinic observation hours. Students also received clinic onboarding presentations and training for new FTC volunteers and employees.

### PhotoVoice

PhotoVoice was developed in the mid-1990s by Caroline Wang and her colleagues to engage communities in community-based participatory research.^14^ PhotoVoice has three main goals: to allow participants to record and reflect on the strengths and issues affecting their community, to promote critical dialogue and increase knowledge about important issues through discussion of photographs, and to reach policy-makers to initiate change.^14^ During the past 20 years, people implemented PhotoVoice in many settings with various populations to raise awareness about and address health disparities.^15^ PhotoVoice as a methodology demonstrates the power of using visual images to engage people in critical thought and discussion about their communities.

### Recruitment and participants

Participants in this study were at least 18 years of age and a current or former patient at FTC. More than 120 patients responded to our recruitment email and 40 interested patients were randomly selected to participate in the PhotoVoice focus groups. Of these, 28 attended the initial orientation and information meeting and 22 submitted photos and captions returning the next week for the focus group discussion. A total of three focus group discussions of 44 photos took place. Participants received an incentive gift card for attending and participating in each focus group; $10 for the first, and $40 for the second.^16^

### Data analysis

In addition to audio recording, each PhotoVoice focus group session had two notetakers. The audio was transcribed and compared with the notes for accuracy. Teams of four to five students began data configuration by familiarizing themselves with the data through multiple readings of the records and then incrementally analyzed data through themes in an initial tagging. At this stage, the entire research team came together to continue tagging. Then these concepts were put into categories, coded, and put into broader themes with representative quotes included.

## Results

### Sociodemographics

The average age of the 28 participants who made up the sample of this study was 30 years (range 19–54). Participants came from 14 different zip codes. The majority of participants identified as white (*n*=15), female (*n*=20), and heterosexual (*n*=13). However, there was a diverse group of race/ethnicities, gender identities, and sexual orientations (Table 1). The demographics of our participants are representative of the larger clinic patient pool, as verified by clinic statistics.

**Table 1.** Characteristics of Sample *n*=28

Gender identity
Female, 20
Male, 2
Transgender, 2
Nonbinary, 2
No answer, 2
Sexual orientation
Bisexual/Pan, 4
Fluid, 2
Heterosexual, 15
Lesbian, 1
Queer, 3
No answer, 3
Race/ethnic identity (self-identified categories)
White, 15
Black, 5
Asian, 1
Hispanic, 3
Filipino/white, 1
Hispanic/white, 2
No answer, 1
Education
Some high school, 2
High school completed, 2
Some college, 4
Currently in college, 5
Associates degree, 1
Bachelor's degree, 11
Master's degree, 3
Household income
<10,000, 3
10–15,000, 2
20–30,000, 3
30–50,000, 3
50,000+, 13
No answer, 4

The major findings summarized hereunder highlight the PhotoVoice Focus Group discussions with FTC patients. Patients repeatedly reference experiences at other clinics where they were required to bear the burden of bureaucratic structures, repeatedly teaching staff and providers how they want to be treated, and facing numerous impediments to receiving the care they need, on their terms. FTC removes these barriers by training its staff to navigate these systems by working with ideologies of equity, in hopes of alleviating the burden on the patients and delivering quality health care. FTC is, in short, exceptional for this reason. Our findings demonstrate five key subthemes under the overarching theme of exceptionalism: community, accessibility, inclusivity, education, and agency.

#### Exceptionalism

**FIG. 1.**
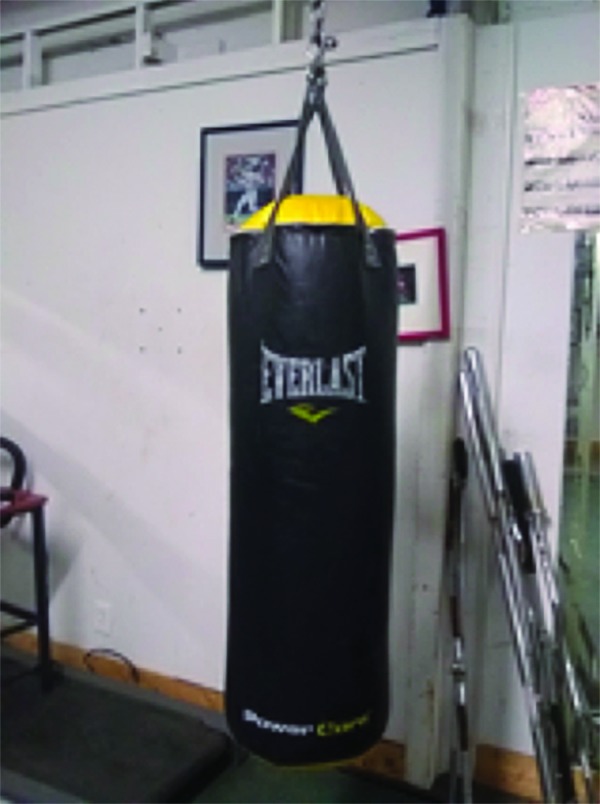
“I am a transgender African American male. This pic here is of a weight room where I work out and practice MMA and bodybuild. I choose Family Tree for therapy because Family Tree is my strength to help me grow and build my body to be the best man I can be. Thank you Family Tree for all your love, help, and care.” (03.03.05)

Figure 1 underscores how patients experience the intentional efforts of the clinic to welcome people of all identities and the positive impact it has on patients.

**FIG. 2.**
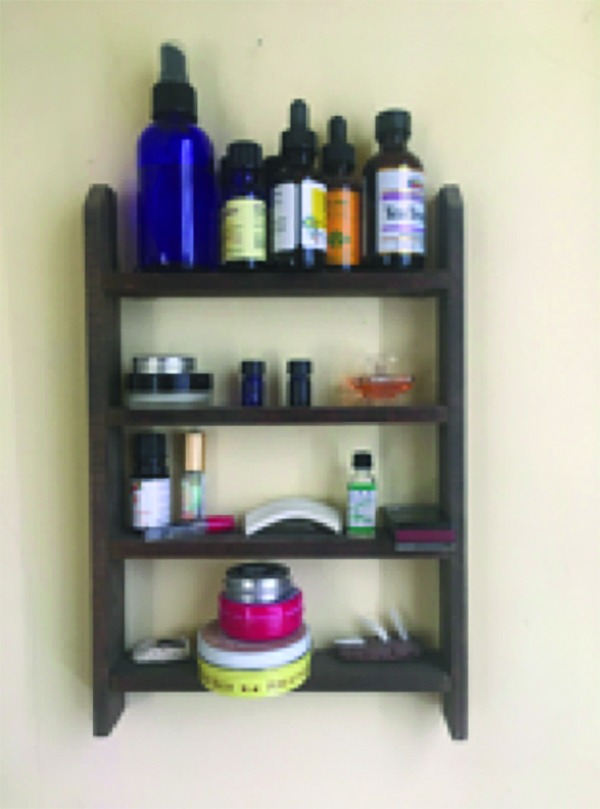
“The first photo attached is of a number of essential oils I have in my home. The first time I went to FTC my gynecologist sprayed a little bit of lavender oil to create a calming environment. This was a huge gesture that was a surprisingly sweet touch and really helped me appreciate the care and attention of the entire staff.” (01.02.08). FTC, Family Tree Clinic.

Figure 2 accentuates an experience of a patient who goes beyond purely medical and emphasizes the impact of FTC's efforts to provide a welcoming and inclusive environment.

Patients shared their experiences at the clinic:

Even before I talk to somebody or they give me specific information I feel more trusting and that makes me better able to ask questions, be more in the present more likely to come receive care when I need it, more likely to follow up (01.01.06).

FTC makes me feel happy to be trans, which isn't how I feel most places. I trust the staff to see me, respect me, and be informed about sexual and reproductive health care issues that affect me. I love my gender when I visit Family Tree. (01.01.06)

Patients provided numerous accounts of the positive impact of FTC's exceptionalism as evidence of their commitment to health equity, reproductive justice, racial oppression, and sex positivity.

#### Community

**FIG. 3.**
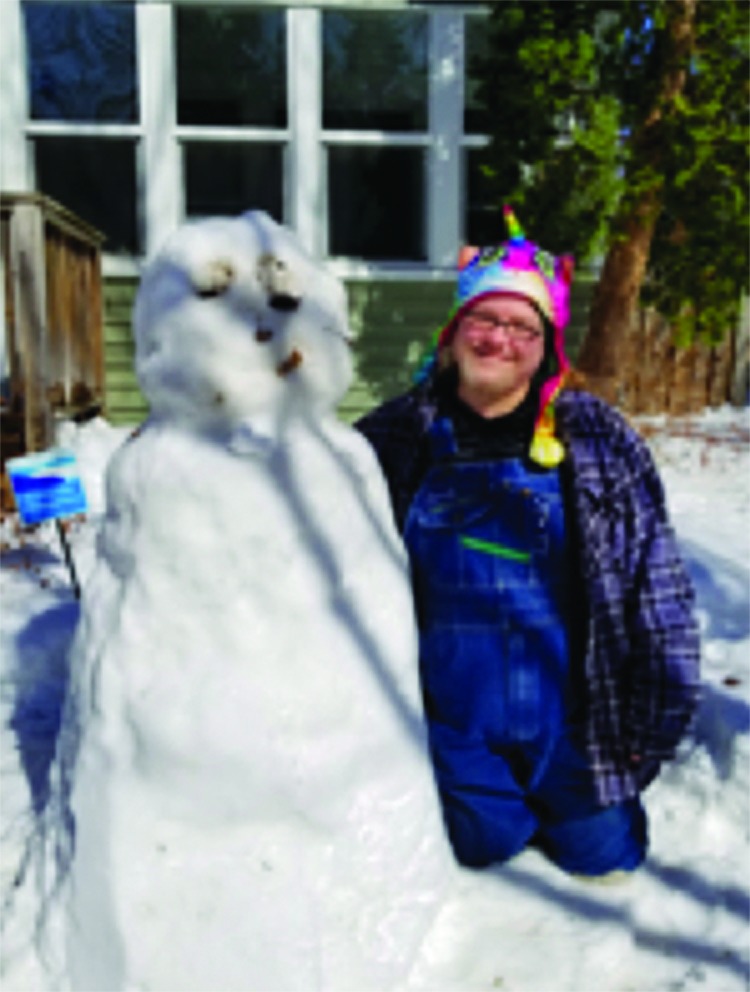
“The clinic uses a lot of open language and not just the clinical terminology. They may use it and expand on it so that you understand, and it's not doctor to doctor but patient to doctor.” (01.03.08) (Image used with permission)

Patients of FTC discussed the pride they feel for their community when receiving care at FTC, Fig. 3.

Patients also emphasize the importance of relationships and person-centered care:

Health is about community. Education, connection and support are integral to maintaining good health. Having a friend or neighbor to walk with, to talk with about what is being experienced physically, socially and emotionally provides wellbeing. What we need to do to have a community around health care, FTC definitely provides that. (02.06.06)

FTC reinforces the importance of centering patients' interests in the care they provide, as representatives and members of the community they serve, to build effective health programs.

#### Accessibility

**FIG. 4.**
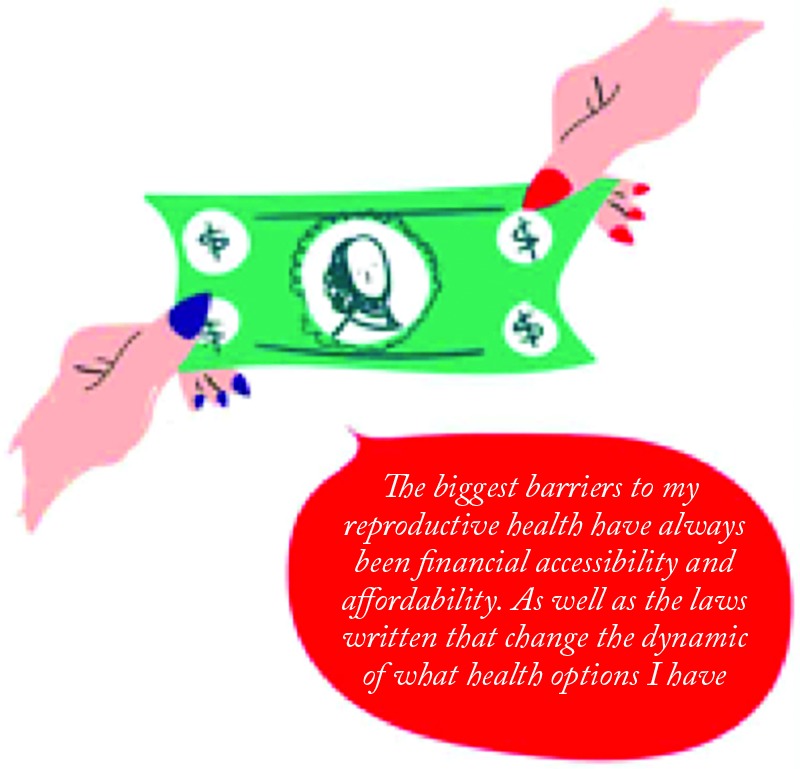
“The biggest barriers to my reproductive health have always been financial accessibility and affordability. As well as the laws written that change the dynamic of what health options I have.” (03.12.08)

Patients identified accessibility at FTC in four ways: location, opportunities for different financial accommodations, hours of operation, and care for different identities, Fig. 4.

Patients are aware of the numerous additional avenues FTC creates to ensure its patients have individualized and quality access to care.

When my health insurance shut me out from coverage for reproductive health care services, FTC welcomed me in with friendly, supportive, open arms and access to their highly recommended services. (03.01.07)

FTC equitably provides resources to its community members and exemplifies this with regard to location, population served, financial accommodations, and medical staff competency.

#### Inclusivity

**FIG. 5.**
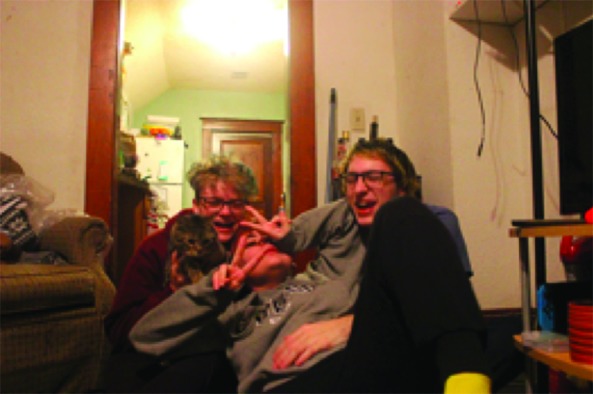
“So, I live with my partner, who is non-binary, and our roommate, who is also non-binary, and our cat. *room laughs* They are my family because my family kicked me out when I started hormones. *participant tears up* So Family Tree's been really an important part of our lives because most places are not super educated about trans topics and Family Tree, at least when I started my transition, was I think the only place that had the informed consent policy, which was important to me because I didn't need a therapist to tell me who I am.” (Image used with permission)

FTC invites people from different backgrounds with their intersecting identities, statuses, and positions in life. At FTC, inclusivity means using correct pronouns, navigating conversations on bodies with understanding, and meeting people where they are at—for example, staff work to provide help and care in patients' preferred language, including ASL, Fig. 5.

Participants indicate FTC is among the community that accepts them: “I came here because I liked that I don't have to always correct when I was seeing a provider or always do that self advocacy” (01.04.02). Others describe the clinic as “Being laid back easy going just, being like a person, no judgment, yeah just real comfortable” (01.08.07). FTC makes people feel welcome, included, and accepted.

#### Education

**FIG. 6.**
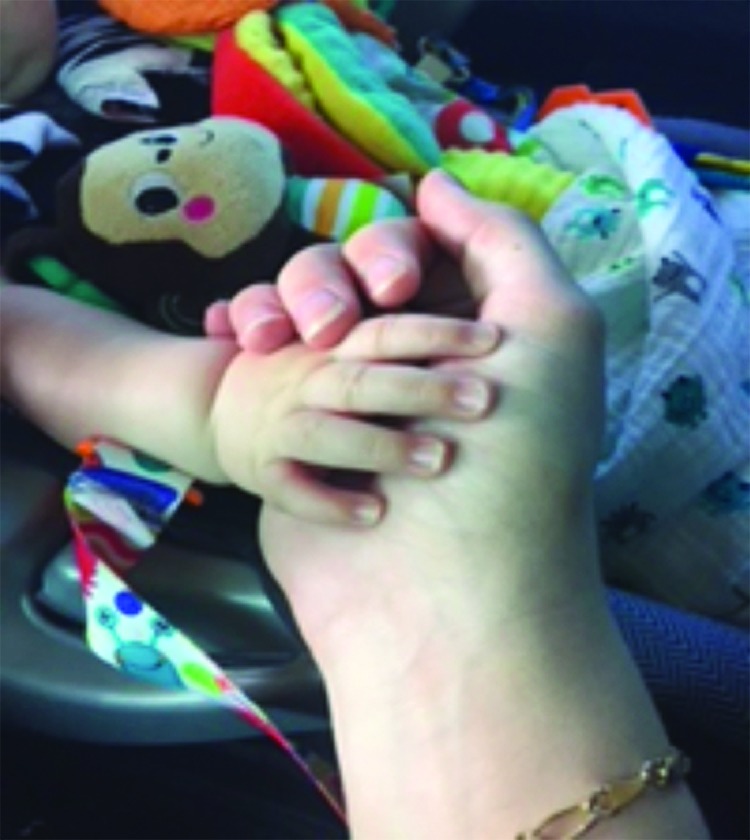
“This is a picture of me and my little nephew…when I became sexually active and I was looking for birth control, FTC was the first place I thought of through my high school and my parents never really talked about safe sex or what to do…And so I was pretty much in the dark on what the norms were about everything. I am already kind of a timid person, it takes me a while to warm up to people. And so I came here looking for birth control and I was kind of surprised by such the welcoming atmosphere and I didn't feel any judgement. I felt like talking right away.” (02.05.04)

Our findings demonstrate two essential ways FTC approaches education. First through its work in community outreach, and second, in the general knowledge of clinic staff. Both the participant observation and focus group findings underscore the noteworthy work of the clinic in the community, Fig. 6.

To continue, participants mention memories of FTC outside of the clinic:

Yeah, I think that they came during the health classes and I think a couple other times. It helped a lot because FTC goes around the grapevine in my school. Everyone knows what it is so they are gonna go there so that if they ever do need medical attention of any kind they'll know at least one name to go to. (02.04.04)

The health professionals at FTC exercise expertise extending beyond other locations participants encountered. Participants describe professionals at other clinics: “It's frustrating when you have to explain medical things to your medical professional” (03.02.03). Patients express frustration that other clinics lack simple competencies for diverse populations. Patients view professionals at FTC as knowledgeable, conscientious, and considerate.

#### Agency and autonomy

**FIG. 7.**
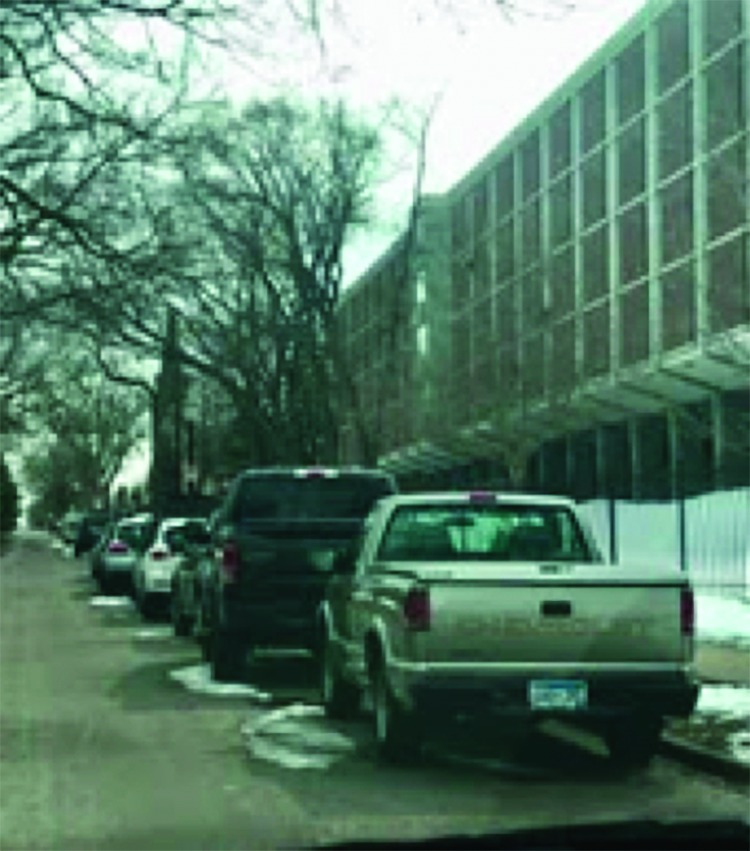
“While it may appear mundane, this picture of my little truck, fondly known as Chance, represents my independence. It is my first car and having a vehicle on campus means I am able to find my own health care providers for the first time in my life…Convenience and confidentiality are really what I wanted, and this picture represents the agency it allows as I am on my own, spreading my wings for the first time.” (03.07.06)

For numerous participants, FTC provided their first opportunity to speak confidently about their needs, without fear or hesitation about repercussions. In previous clinical encounters, participants explain feeling talked down to, as if they were not given all of the options, guided to make a decision they did not want to, or pressured to conform to a standard, Fig. 7.

Patients extended the discussion of how FTC helps:

[I started to] feel confident about my sexual health and about my health in general when I started going to Family Tree. I felt really confident about my decisions and what my options were. (03.06.02)

Another participant identified the importance of receiving care without disruption and judgment:

The informed consent hormone therapy is very important to me not because I use it, but because my girlfriend does. And it's important to me that she gets that. So it's just very nice to have the welcoming environment and the ability to just show up and be like ‘Hello, I am trans. I need hormones.’ Instead of jumping through as many bureaucratic hoops as other places would make you go through. (02.03.03)

Overall, the environment and commitment of FTC to health equity provide the space and liberty to get services without contempt.

The mentioned themes address the exceptional aspects of the health services participants receive at FTC. Overall, the barriers people face in achieving their health goals relate to two overarching categories: first, the absence of the mentioned themes in practice, and the presence of the second set of themes related to trade-offs and obstacles that describe structural and systemic hurdles participants face that do not directly relate to the services they receive at FTC.

## Discussion

This research reveals how integrating health equity as a tool for providing care produces equitable and positive outcomes. Patients of the FTC highlighted the barriers to achieving their goals for their health, all of which were located outside the clinic's purview, yet identified how the clinic's specific inclusive and equity-focused practices work to mitigate these barriers. FTC's health equity-centered approach moves beyond simply recognizing the social determinants of health and the impact of social structures. Their intentional core values rooted in reproductive justice, LGBTQ+ core competencies, sex positivity, and intersectionality directly create actionable health equity outcomes.

Patients of the FTC articulate that the aims of the clinic to address reproductive justice and intersectionality by serving the reproductive and sexual health needs of marginalized and vulnerable communities who experience oppression make a difference to patients. The clinic provides patient-centered services where patients have a say in determining their needs: in terms of what type of care they receive, where they receive it, how they receive it, and in which ways they can pay for that care. Patients discussed FTC as a place of refuge when it comes to sex and health. Recognition and validation of one's gender identity are necessary for sex positivity.

It is clear patients appreciate FTC's efforts to approach patient care by addressing the heterogeneous health needs and disparities of LGBTQ+ communities with attention to intersectionality.^5–7^ For example, one patient mentioned “The gender checkboxes on the intake form” are demonstrative of how FTC respects “the pronouns I entered on the form and didn't just treat [pronouns] like a formality …”(02.03.03). Another pointed out: “It's frustrating when you have to explain medical things to your medical professional” (03.02.03). Patients express frustration that other clinics lack simple competencies for diverse populations of people. Patients view professionals at FTC as knowledgeable, conscientious, and considerate.

FTC is a clinic that operates from a model where they empower, and support their patients to be healthy and stay healthy. For example, the use of gender-neutral pronouns in health care facilities is yet to be adopted as a standardized approach to addressing patients; however, patients identified that by not referring to them with their appropriate gender pronouns, or affirming their beliefs about themselves, created unsafe and tense environments that leads to them having to sacrifice parts of their identities to receive any care. In response, FTC embedded into its mission statement and practices to respect patient's gender pronouns. This clearly illustrates the need to integrate our communities' needs into our systems, practices, and policies to deliver the most appropriate, high-quality, and effective care we can. It signifies the urgency to retire treating patients as standardized beings and instead embrace the complexity and fluidity of human beings in the health care system. Intersectionality affirms this urgency and states that patients should not have to maneuver themselves to fit into overly simplistic boxes that ignore the complexities of their social life as a byproduct of systems of power that are intrinsically connected to a patient's identity.

## Conclusion

We asked patients of FTC two questions: Why do you choose to come to FTC and What stands in the way of achieving your goals for your health? The resulting PhotoVoice focus group discussions highlight areas other clinics can incorporate into their systems and structures to improve patient experience. Using intersectionality as an analytical tool not only enhances our ability to conceptualize our society, but it also draws clear areas of improvement in the delivery of care.^17,18^ One key area of improvement is in improving and evolving clinical practices to include the language and intellectual wisdom of social justice movements, and two is to address systemic forces of power that are present in physician–patient relationships. Traditional biomedical models fail to transcend into the vast dimensions of human identities and experiences and largely focus on perceived individualistic behaviors of people as reasons for their less-than-favorable health outcomes. These models have not embraced the extensive, illuminating, and data-driven results that researchers in critical race studies, public health, psychology, history, and other converging academic disciplines have addressed and established.

## Abbreviation Used

FTC: Family Tree Clinic
